# Heterogeneity of HTLV-1 proviral integration sites and internal structures in the ATL cell line MT-1

**DOI:** 10.1007/s13577-026-01385-1

**Published:** 2026-04-30

**Authors:** Misaki Izaki, Yuki Hashikura, Kunihiko Umekita, Kazumi Umeki, Taiga Miyazaki

**Affiliations:** 1 Clinical Laboratory, University of Miyazaki Hospital, University of Miyazaki, Miyazaki, Japan; 2 Division of Respirology, Rheumatology, Infectious Diseases, and Neurology, Department of Internal Medicine, Faculty of Medicine, University of Miyazaki, 5200 Kihara, Kiyotake, Miyazaki, 889-1692 Japan

**Keywords:** Human T-cell leukemia virus type 1, Leukemogenesis, PCR

## Abstract

Human T-cell leukemia virus type 1 (HTLV-1) causes adult T-cell leukemia (ATL). The ATL-derived cell line MT-1 is used to study HTLV-1 biology and leukemogenesis. However, the proviral integration sites and internal structures of HTLV-1 are not comprehensively characterized in MT-1 cells. We analyzed two independently maintained MT-1 cell lines (MT-1 J and MT-1 M) using long PCR, quantitative PCR-based proviral load analysis, inverse long PCR, inverse PCR, integration site-specific PCR, direct sequencing, and Rapid Amplification of Integration Site without Interference by Genomic DNA contamination. Long PCR and proviral load analyses demonstrated that MT-1 cells harbor multiple full-length and defective HTLV-1 proviruses. MT-1 J cells exhibited reduced proviral load in the *pX* region, suggesting the presence of *pX*-lacking proviruses. Inverse long PCR and inverse PCR revealed at least five proviral integration sites in MT-1 J cells; MT-1 M cells contained three. Site-specific PCR confirmed the differential preservation of integration sites. Sequence analysis revealed two full-length proviruses and three type I defective proviruses with distinct internal deletions, including an unreported provirus with a large deletion encompassing *pX*. Rapid Amplification of Integration Site without Interference by Genomic DNA contamination identified major proviral clones shared between MT-1 J and MT-1 M cells, while revealing differences in clone frequencies and minor integration sites. The MT-1 cell line is a polyclonal population containing multiple full-length and defective HTLV-1 proviruses. Its proviral composition can change during long-term in vitro passaging. This heterogeneity should be considered when interpreting results obtained using MT-1 cells in HTLV-1 and ATL research.

## Introduction

Human T-cell leukemia virus type 1 (HTLV-1) is the etiological agent of adult T-cell leukemia (ATL) [1]. In addition to ATL, HTLV-1 is associated with a spectrum of inflammatory and neurological diseases, including HTLV-1-associated myelopathy/tropical spastic paraparesis and HTLV-1-associated uveitis [2–4]. After infecting human T cells, HTLV-1 integrates into the host genome as a provirus. Not all HTLV-1 carriers develop ATL; it is estimated that 3%–5% of infected individuals progress to ATL after a prolonged latent period [5].

HTLV-1-infected cell lines and ATL-derived cell lines have been extensively used as experimental cell lines to investigate the mechanism of viral infection and the pathogenesis of ATL. Recent studies have demonstrated that viral enhancers within the HTLV-1 provirus can aberrantly regulate the transcription of host genes near proviral integration sites, thereby contributing to leukemogenesis [6]. The HTLV-1 regulatory gene *tax* and accessory gene *HBZ* play central roles in promoting the proliferation and malignant transformation of infected cells [7, 8]. These findings underscore the importance of characterizing the genomic integration sites, copy numbers, and structural features of HTLV-1 proviruses to understand ATL pathogenesis.

We previously analyzed the HTLV-1-infected cell line MT-2 and demonstrated that a single MT-2 cell line harbors at least 11 distinct HTLV-1 proviral integration sites and the composition of the integration site differs between MT-2 cell lines maintained in different laboratories [9]. The MT-2 cell lines exhibited differences in the production levels of cytokines, such as IFN-γ, TNF-α, and TNF-β [10]. These findings suggest that long-term in vitro passaging and culture conditions alter both the proviral integration landscape and cellular phenotypes. However, it is unclear whether the diversity in proviral composition is unique to the MT-2 cell line or represents a general feature of ATL-derived cell lines. Heterogeneity in other widely used ATL cell lines would indicate significant misinterpretation of previously reported results.

The ATL cell line MT-1, originally established by Miyoshi et al., is one of the most representative and widely used cell lines in HTLV-1 and ATL research. PCR-in situ hybridization has revealed that MT-1 cells harbor 1–3 copies of HTLV-1 proviral DNA per cell [11]. However, comprehensive analyses of proviral integration sites and the internal structures of individual proviruses in MT-1 cells have not been reported. Determining whether the proviral heterogeneity and passaging-associated changes in MT-2 cells also occur in MT-1 cells is essential for interpreting results and designing experiments using the MT-1 cell line.

In this study, we analyzed the HTLV-1 proviral integration sites and internal structures in the MT-1 cell line, which is used as a standard experimental cell line in ATL and HTLV-1 research. By comparing independently maintained MT-1 cell lines, we aimed to 1) determine whether MT-1 exhibits heterogeneity in proviral composition similar to that in MT-2 cells and 2) assess the impact of long-term in vitro passaging on the composition of proviral integration sites.

## Material and methods

### Cell lines and DNA extraction

The ATL cell line MT-1 was purchased from the JCRB Cell Bank, National Institutes of Biomedical Innovation, Health and Nutrition (cell no. JCRB1209, lot 12,172,007) and used as the primary material for analysis (hereafter referred to as MT-1 J) [12]. To evaluate differences in proviral integration sites, an MT-1 cell line (established by Dr. Miyoshi) and maintained by serial passage in the laboratory of Dr. Morishita at the University of Miyazaki was used for comparison (hereafter referred to as MT-1 M). The HTLV-1-infected cell line MT-2 (cell no. JCRB1210, lot 09142007) and the HTLV-1-negative T-cell line Jurkat (cell no. RBC3052) were used as positive and negative controls, respectively.

All cell lines were cultured in RPMI-1640 medium (Nacalai Tesque, Kyoto, Japan) supplemented with 10% fetal bovine serum, 5 μg/mL penicillin, 5 μg/mL streptomycin, and 10 μg/mL neomycin. Genomic DNA was extracted from cultured cells using the QIAamp DNA Blood Mini Kit (QIAGEN, Tokyo, Japan), according to the manufacturer’s protocol.

### Long PCR

Genomic DNA (100 ng) extracted from MT-1 J and MT-1 M cells was used as a template for long-range PCR to amplify near full-length HTLV-1 proviral genomes. PCR was performed using primers annealed to both ends of the provirus: HTLV-647F (5′-GTTCCACCCCTTTCCCTTTCATTCACGACTGACTGC-3′) and HTLV-8345R (5′-GGCTCTAAGCCCCCGGGGGATATTTGGGGCTCATGG-3′). After initial denaturation at 98 °C for 20 s, 32 cycles of PCR amplification were performed with denaturation at 98 °C for 10 s, annealing at 68 °C for 20 s, and extension at 72 °C for 7 min. PCR products were analyzed by electrophoresis on a 0.8% agarose gel.

### Quantification of HTLV-1 proviral load and detection of defective proviruses in three genomic regions

Quantification of HTLV-1 proviral load (PVL) and detection of defective proviruses were performed according to Ueno et al. [13] using real-time PCR targeting the LTR-*gag*, *gag*, and *pX* regions, with the human albumin gene used as a single-copy reference control. PCR amplification, fluorescence detection, and standard curve generation were performed as described by Ueno et al. PVL was calculated as the number of HTLV-1 copies per two copies of the human albumin gene, corresponding to one cell.

### Detection of HTLV-1 proviral integration sites using inverse long PCR

HTLV-1 proviral integration sites in MT-1 J and MT-1 M cells were identified using inverse long PCR (IL-PCR) according to Hashikura et al. and Etoh et al. [9, 14]. Briefly, 3.2 μg of genomic DNA extracted from MT-1 J and MT-1 M cells was digested with *Eco*RI, followed by self-ligation using T4 DNA ligase to generate circular DNA. The circular DNA was then linearized with *Mlu*I, and the resulting fragments were cloned into the pGEM-T Easy vector and transformed into *Escherichia coli* JM109. Recombinant clones were screened by colony PCR to identify inserts containing HTLV-1 proviral junctions. Positive clones were sequenced using the BigDye Terminator v3.1 Cycle Sequencing Kit and analyzed on an Applied Biosystems 3500 Genetic Analyzer. The resulting sequences were aligned to the human genome using BLAT (UCSC Genome Browser) to determine host genomic regions flanking integrated HTLV-1 proviruses.

### Detection of HTLV-1 proviral integration sites by inverse PCR

HTLV-1 proviral integration sites in MT-1 J and MT-1 M cells were analyzed using inverse PCR (I-PCR) according to Hashikura et al. and Takemoto et al. [9, 15]. Briefly, genomic DNA was digested with *Sau*3AI or *Alu*I, self-ligated, and subsequently digested with *Sac*II. The resulting DNA was PCR-amplified using 3′ LTR-specific primers, followed by semi-nested PCR. The amplified products were then cloned, sequenced, and aligned to the human genome using BLAT to identify proviral integration sites.

### Integration site-specific PCR

To compare proviral integration sites identified in MT-1 J with those in MT-1 M cells, integration site-specific PCR was performed using MT-1 J as the reference. Purified genomic DNA was used as template for PCR amplification with primer pairs consisting of an HTLV-1-specific primer, HTLV-9014F (5′-AGCCCATCCTATAGCACTCTC-3′), and a primer specific to the host genomic sequences flanking each integration site. PCR products were analyzed using electrophoresis on a 1.0% agarose gel to confirm site-specific amplification.

### Comparison of proviral internal structures between MT-1 J and MT-1 M cells and the HTLV-1 reference sequence

To examine the internal structures of HTLV-1 proviruses integrated into the host genome, genomic DNA extracted from MT-1 J and MT-1 M cells was used as a template for PCR. To analyze the 5′ LTR-proximal region, PCR was performed using the HTLV-1-specific primer HTLV-7002R in combination with primers specific to the MT-1 host genomic sequences flanking the 5′ LTR of each integration site (Table 1). Similarly, to amplify the 3′ LTR-proximal region, PCR was performed using the HTLV-1206CF primer in combination with primers specific to host genomic sequences adjacent to the 3′ LTR (Table 1).

**Table 1:** Primer sets used to analyze the internal structure of the human T-cell leukemia virus type 1 (HTLV-1) provirus

Integration site	Primer name	Sequence
Site A	MT-1-Chr11F	5′- CCATAACAGATGGCCTTCACATTTAGGAAG-3′
	HTLV-7002R	5′-AGTATTTGAAAAGGAAGGAAGAGGAGAAGGCA-3′
	MT-1-Chr11R	5′- AAGGCAAAATTCAAGCCCAAAGTGGC-3′
	HTLV-1206CF	5′- AAGTCCTTCCAGTCATGCACCCACATGGTG-3′
Site B	MT-1-Chr18F	5′- ATGGGGTTTCACCATGTTGGTC-3′
	HTLV-7002R	5′-AGTATTTGAAAAGGAAGGAAGAGGAGAAGGCA-3′
	MT-1-Chr18R	5′- TGGGAGTTCGAGACCAGTGTTGTG-3′
	HTLV-1206CF	5′- AAGTCCTTCCAGTCATGCACCCACATGGTG-3′
Site C	MT-1-Chr1F	5′- TACATGGTTTCACATGCCTGGTAGGC-3′
	HTLV-7002R	5′-AGTATTTGAAAAGGAAGGAAGAGGAGAAGGCA-3′
	MT-1-Chr1R	5′- CCAGTGCACTACAGAGTGAGACTCCGTCTC-3′
	HTLV-1206CF	5′- AAGTCCTTCCAGTCATGCACCCACATGGTG-3′
Site D	MT-1-Chr5F	5′- CTCCCAGGTAATCCTCTTACTTGTCAC-3′
	HTLV-7002R	5′-AGTATTTGAAAAGGAAGGAAGAGGAGAAGGCA-3′
	MT-1-Chr5R	5′- TCCCATTCCTTCACCCCCTCATGCAAAG-3′
	HTLV-1206CF	5′- AAGTCCTTCCAGTCATGCACCCACATGGTG-3′
Site E	MT-1-Chr10F	5′- GGCAGAAACATGGGCTTGCAAATGTAG-3′
	HTLV-7002R	5′-AGTATTTGAAAAGGAAGGAAGAGGAGAAGGCA-3′
	MT-1-Chr10R	5′- CCTGAATGACAGAGCAAGACCCTGTTTC-3′
	HTLV-1206CF	5′- AAGTCCTTCCAGTCATGCACCCACATGGTG-3′

The PCR products were directly sequenced. The sequences were compared with the HTLV-1 reference sequence (DDBJ/EMBL/GenBank accession no. J02029) to assess the presence and extent of internal deletions within the proviral genomes.

### Comprehensive analysis of HTLV-1 proviral integration sites using rapid amplification of integration site without interference by genomic DNA contamination

Comprehensive identification of HTLV-1 proviral integration sites was performed using Rapid Amplification of Integration Site without Interference by Genomic DNA contamination (RAISING)—a next-generation sequencing-based approach that enables high-throughput detection of proviral integration sites within the host genome [16]. Single-stranded DNA containing the junction between the HTLV-1 provirus and host genomic DNA was synthesized using HTLV-1-specific primers. Polynucleotide tails and adapter sequences were added to the 3′ end of the single-stranded DNA. Double-stranded DNA was generated using a provirus-specific primer and an oligo(dT) primer, followed by high-throughput sequencing to identify proviral integration sites. RAISING analysis was performed by LSI Medience Corporation (Tokyo, Japan).

### Statistical analysis

Statistical comparisons among the three regions (LTR-*gag*, *gag*, and *pX*) were performed using one-way ANOVA followed by Tukey’s multiple comparisons test. A *P* value of < 0.05 was considered statistically significant. All analyses were performed using SPSS Statistics version 20 (IBM, Chicago, IL, USA).

## Results

### Detection of full-length and defective HTLV-1 proviruses using long PCR

**Fig. 1:**
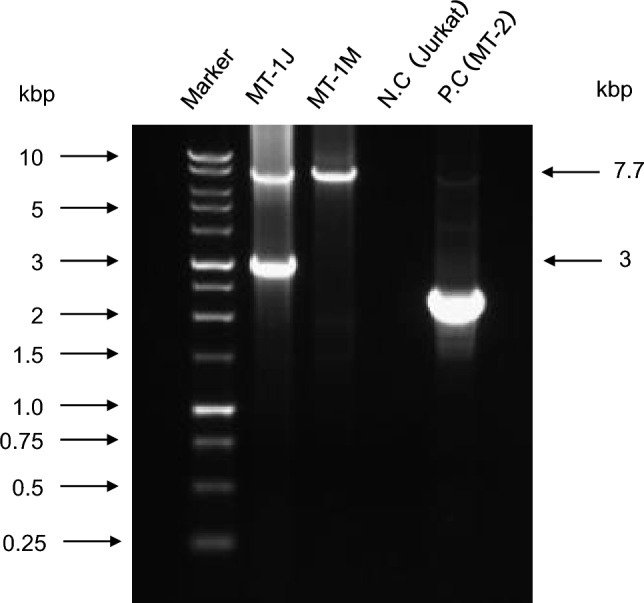
Detection of full-length and defective human T-cell leukemia virus type 1 (HTLV-1) proviruses using long PCR. Long PCR products were separated by electrophoresis on a 0.8% agarose gel. In MT-1 J cells, two bands were detected: a ~ 7.7-kb band corresponding to the full-length HTLV-1 provirus and a ~ 3.0-kb band consistent with a defective provirus harboring a large internal deletion. By contrast, MT-1 M cells predominantly exhibited a single band of ~ 7.7 kb, corresponding to the full-length provirus. No amplification product was detected in HTLV-1-negative control Jurkat cells. HTLV-1-positive control MT-2 cells showed bands corresponding to the full-length provirus (~ 7.7 kb) and the defective provirus (~ 2.4 kb), as reported [9]

Nearly full-length HTLV-1 proviruses integrated into the cellular genome were amplified by long PCR. In the MT-1 J cell line, in addition to a band of ~ 7.7 kb that corresponded with full-length provirus, an additional band of ~ 3.0 kb was detected, which is consistent with a provirus harboring an internal deletion of ~ 4.7 kb. By contrast, the MT-1 M cell line predominantly exhibited a single band of ~ 7.7 kb, corresponding to full-length provirus (Fig. 1). Thus, MT-1 J cells contain a mixture of HTLV-1 proviruses with different internal structures.

### Proviral load analysis in three genomic regions and detection of defective proviruses

**Fig. 2:**
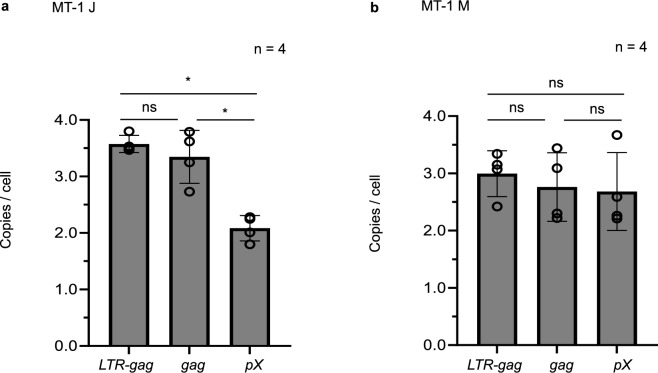
Proviral load analysis of three human T-cell leukemia virus type 1 (HTLV-1) genomic regions in MT-1 cells. HTLV-1 proviral copy numbers in the LTR-*gag*, *gag*, and *pX* regions were quantified by real-time PCR and normalized to two copies of the gene for cellular *albumin*. **a** In MT-1 J cells, the copy numbers were 3.57, 3.35, and 2.08 for the LTR-*gag*, *gag*, and *pX* regions, respectively. The *pX* region had a significantly lower copy number than the LTR-*gag* and *gag* regions (*P* < 0.05). **b** In MT-1 M cells, the copy numbers were 3.0, 2.8, and 2.7 for the LTR-*gag*, *gag*, and *pX* regions, respectively, with no significant differences among the three regions. Data are presented as mean ± standard deviation (SD) of four independent biological experiments (*n* = 4). Error bars represent the SD. Statistical comparisons were performed using one-way ANOVA followed by Tukey’s post hoc test

To evaluate HTLV-1 proviral copy numbers and the presence of internal deletions, PVL was determined using real-time PCR targeting three HTLV-1 genomic regions (LTR-*gag*, *gag*, and* pX*) with the human albumin gene used as the single-copy reference control. In MT-1 J cells, copy numbers were 3.57, 3.35, and 2.08 for LTR-*gag*, *gag*, and *pX* regions, respectively. The *pX* region had a lower copy number than the LTR-*gag* and *gag* regions (Fig. 2a). By contrast, in MT-1 M cells, copy numbers were 3.0, 2.8, and 2.7 for LTR-*gag*, *gag*, and *pX* regions, respectively, with no marked differences among the three regions (Fig. 2b). These findings suggest that MT-1 J cells harbor HTLV-1 proviruses lacking the *pX* region and further support the presence of multiple proviruses with distinct internal structures. In MT-1 J cells, the copy number of the *pX* region was significantly lower than those of the LTR-*gag* and *gag* regions (*P* < 0.05) (Fig. 2a). In contrast, no significant differences in copy number were observed among the three regions in MT-1 M cells (Fig. 2b).

### Identification of HTLV-1 proviral integration sites in MT-1 cell lines

To identify the host genomic integration sites of HTLV-1 proviruses in MT-1 J and MT-1 M cells, we performed IL-PCR and I-PCR. IL-PCR detected two integration sites (A and B) in MT-1 J cells; only site A was detected in MT-1 M cells (Table 2). By contrast, I-PCR identified four integration sites (B–E) in MT-1 J cells; only sites C and D were detected in MT-1 M cells (Table 2).

**Table 2:** Integration sites identified in the MT-1 J cell line using inverse long PCR (IL-PCR) and inverse PCR (I-PCR)

Site of integration	Chromosome	IL-PCR		I-PCR	
		MT-1 J	MT-1 M	MT-1 J	MT-1 M
A	11(p15.3)	○	○	−	−
B	18(q22.3)	○	−	○	−
C	1(p36.31)	−	−	○	○
D	5(p13.1)	−	−	○	○
E	10(q11.23)	−	−	○	−

○ indicates identified integration site

Taken together, MT-1 J cells harbor at least five distinct HTLV-1 proviral integration sites, some of which are not retained in MT-1 M cells.

### Validation of proviral integration sites by site-specific PCR

**Fig. 3:**
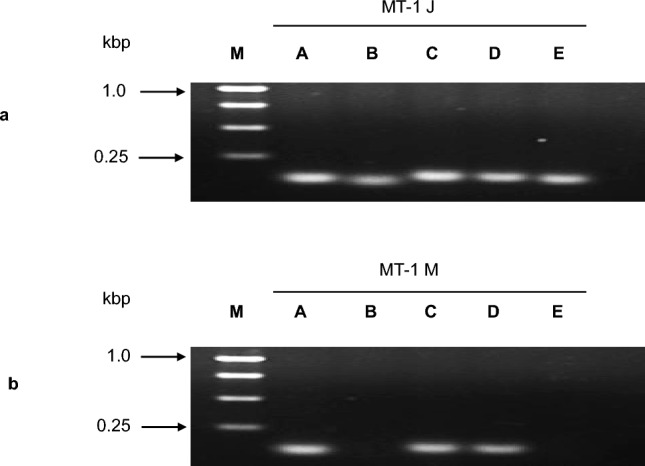
Validation of human T-cell leukemia virus type 1 (HTLV-1) proviral integration sites by integration site-specific PCR. PCR was performed using the HTLV-1-specific primer HTLV-9014F in combination with primers specific to host genomic sequences flanking each of the five identified proviral integration sites (A–E). PCR products were analyzed by agarose gel electrophoresis. **a** in MT-1 J cells, clear amplification products were detected for all five integration sites. **b** in MT-1 M cells, amplification products were detected for integration sites A, C, and D. No products were detected for sites B and E. M, molecular size marker

To confirm the presence of the proviral integration sites identified by IL-PCR and I-PCR, integration site-specific PCR was performed using primer sets specific to the host genomic sequences flanking each integration site in combination with provirus-specific primers. In MT-1 J cells, amplification products were detected for all five integration sites (sites A–E) (Fig. 3a). By contrast, MT-1 M cells yielded amplification products only for sites A, C, and D; no products were detected for sites B and E (Fig. 3b). These results confirm that a subset of the proviral integration sites identified in MT-1 J cells is not preserved in MT-1 M cells.

### Comparison of proviral internal structures with the HTLV-1 reference sequence

**Fig. 4:**
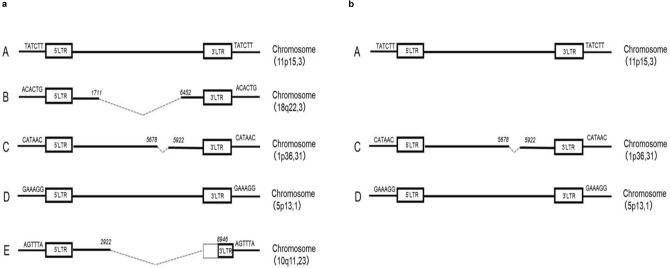
Internal structures of human T-cell leukemia virus type 1 (HTLV-1) proviruses integrated in MT-1 cells. Schematic representation of the internal structures of HTLV-1 proviruses determined by direct sequencing. **a** In MT-1 J cells, full-length proviruses were integrated at sites **A** (11p15.3) and **D** (5p13.1). A defective provirus with an internal deletion spanning nucleotide positions 1712–6451 was integrated at site **B** (18q22.3) and a smaller deletion spanning positions 5679–5921 was detected at site **C** (1p36.31). At site **E** (10q11.23), a large internal deletion spanning positions 2923–8945, including the *pX* region, was identified. Dashed lines indicate deleted regions. **b** In MT-1 M cells, the proviral sequences detected at sites A, C, and D were identical to the corresponding proviruses identified in MT-1 J cells

The internal structures of HTLV-1 proviruses integrated at each identified site in MT-1 J cells were analyzed by direct sequencing and compared with the HTLV-1 reference sequence (J02029). Among the five integration sites, two harbored full-length HTLV-1 proviruses and three contained proviruses with internal deletions (Fig. 4a).

Specifically, full-length proviruses were integrated at sites A and D. At site B, we detected a provirus with an internal deletion of ~ 4.7 kb (nucleotide positions 1712–6451), consistent with the ~ 3.0 kb amplification product observed through long PCR (Fig. 1). At site C, a smaller internal deletion of ~ 0.24 kb (nucleotide positions 5679–5921) was identified. The provirus integrated at site E exhibited a large internal deletion of ~ 6.0 kb spanning nucleotide positions 2923–8945, including *pX*. This finding was consistent with the reduced copy number of *pX* regions detected by quantitative PCR (Fig. 2). The proviral sequences integrated at the three sites detected in MT-1 M cells (sites A, C, and D) were identical to the corresponding proviral sequences identified in MT-1 J cells (Fig. 4b).

### Comprehensive analysis of HTLV-1 proviral integration sites using RAISING

A comprehensive analysis of HTLV-1 proviral integration sites was performed using RAISING. Of the five integration sites identified by IL-PCR and I-PCR, four (A–D) were detected by RAISING. Several novel integration sites that had not been detected by IL-PCR or I-PCR were identified. Integration sites A, C, and D, which were detected by both IL-PCR and I-PCR, were commonly identified in MT-1 J and MT-1 M cells by RAISING and ranked among the top four most frequent integration sites. By contrast, integration site B ranked third in MT-1 J cells, accounting for 17.1% of the total reads, and 33rd in MT-1 M cells, with a markedly lower frequency of 0.01% (Table 3). Integration site E was not detected by RAISING. RAISING identified several integration sites on the Y chromosome that were not detected by IL-PCR or I-PCR (Table 3).

**Table 3:** Top 8 detected integration sites identified through rapid amplification of integration site without interference by genomic DNA contamination (RAISING)

MT-1 J cells						MT-1 M cells					
Site of integration	Chromo some	Position	Read count	Proportio*n* (%)	Gene ID	Site of integration	Chromo some	Position	Read count	Proportio*n* (%)	Gene ID
C	1	6,605,437	12,575	21.3	KLHL21	C	1	6,605,437	14,079	24.6	KLHL21
A	11	12,364,901	10,486	17.8	MICAL2-PARVA	A	11	12,364,901	8285	14.5	MICAL2-PARVA
B	18	74,524,796	10,075	17.1	CNDP2-CNDP1	D	5	40,009,815	6983	12.2	LINC00603
D	5	40,009,815	8244	14.0	LINC00603		Y	56,674,654	6960	12.2	PARP4P1-CTBP2P1
	Y	56,674,654	8091	13.7	PARP4P1-CTBP2P1		1	125,179,284	6271	11.0	LINC01691-AC242852.1
	Y	56,694,487	5278	8.9	PARP4P1-CTBP2P1		Y	56,694,487	4982	8.7	PARP4P1-CTBP2P1
	Y	56,683,791	1846	3.1	PARP4P1-CTBP2P1		1	143,216,680	2622	4.6	LINC01691-AC242852.1
	Y	122,588,252	280	0.5	LINC01691-AC242852.1		1	143,193,711	2228	3.9	LINC01691-AC242852.1

## Discussion

We analyzed the HTLV-1 proviral integration sites and internal structures in the ATL cell line MT-1 in detail. Our findings revealed that MT-1 is not a monoclonal population harboring a single proviral configuration, but rather a polyclonal population containing multiple HTLV-1 proviruses.

Long PCR and PVL quantification revealed that MT-1 cells harbor not only full-length HTLV-1 proviruses, but also defective proviruses. In MT-1 J cells, the PVL values for the LTR-*gag*, *gag*, and *pX* regions were 3.57, 3.35, and 2.08 copies per cell, respectively. In MT-1 M cells, we detected multiple proviral copies. These findings indicate that multiple HTLV-1 proviruses are integrated per cell in the MT-1 cell line. Although the proviral copy number per cell differed from that reported in the MT-2 cell line, the presence of multiple proviruses is consistent with previous observations in MT-2 cells [9]. By contrast, HTLV-1-infected cells derived from carriers or patients with ATL typically harbored a single proviral copy per cell [17]. This discrepancy suggests that multiple proviral infections are suppressed in vivo; alternatively, cells harboring multiple proviruses are selectively eliminated by host immune surveillance. Consistently, Yamamoto et al. reported multiple proviral copies per cell in an HTLV-1-infected cell transplantation model using immunodeficient NOG mice [18]. Thus, in the absence of immune pressure, such as that in the MT-1 cell line, multiple HTLV-1 proviruses may coexist. In addition, the lower PVL observed for the *pX* region compared with the LTR-*gag* and *gag* regions in MT-1 J cells suggests the presence of proviruses that lack the *pX* region.

Although IL-PCR and I-PCR identified five proviral integration sites in MT-1 J cells, PVL did not reach five copies per cell. This discrepancy may be explained by clonal heterogeneity and unequal clonal representation, as supported by the RAISING analysis showing variable clonal frequencies, indicating that not all integration sites contribute equally to the overall proviral load due to differences in clonal abundance. Comparison with MT-1 M cells, which were passaged independently in a different laboratory, suggests that the clonal composition established at early stages of infection changed during long-term in vitro passaging.

Analysis of proviral internal structures revealed that MT-1 J cells harbor two full-length proviruses and three type I defective proviruses with internal deletions. The provirus integrated at site B, which contains an ~ 4.7-kb internal deletion, lacks the second exon of *tax*, indicating that Tax expression from this provirus is structurally impossible. By contrast, the defective provirus at site C retains regions encoding *tax* and *HBZ*, suggesting that *tax* and *HBZ* mRNAs are expressed. A subset of proviruses identified in MT-1 cells lacks the *tax* or *pX* regions, which may influence Tax and/or HBZ expression and thereby affect cellular phenotypes. Because this study did not directly assess viral RNA or protein expression, the potential effects of defective proviruses on Tax and HBZ expression remain speculative and are inferred from proviral structure. Nevertheless, the observed proviral heterogeneity raises the possibility that structural variation among proviruses may contribute to functional differences among MT-1 sublines. Taken together, full-length proviruses and partially defective proviruses retaining *tax* and/or *HB*Z regions are likely to contribute to viral gene expression, whereas severely deleted proviruses are less likely to be transcriptionally active. The provirus integrated at site E exhibited a large internal deletion of ~ 6.0 kb encompassing the *pX* region; this proviral structure has not been reported and was identified in this study. Kuramitsu et al. reported that the *pX* region is generally preserved in proviruses derived from patients with HTLV-1, and this feature underlies the widespread use of the *pX* region as a target for PVL quantification [19]. Therefore, the provirus at site E would not be detected by quantitative PCR assays targeting the *pX* region. Because this provirus lacks both *tax* and *HBZ*, it is unlikely to produce viral proteins independently. Consequently, full-length proviruses at sites A and D, as well as type I defective provirus at site C, may serve as sources of viral protein expression in MT-1 J cells. In MT-2 cells, identically deleted proviruses have been reported to integrate into six different chromosomes; however, such proviruses were not detected in MT-1 cells in the present analysis. Similarly, type 2 defective proviruses previously described in MT-2 were also absent in MT-1, suggesting differences in the types of defective proviruses present between these cell lines [9]. These findings indicate that the proviral composition of MT-1 may differ from that of other ATL-derived cell lines.

Defective proviruses have been reported in 30%–50% of all ATL cases [20–22], and the coexistence of full-length and defective proviruses in MT-1 cells is consistent with other observations [23]. However, the presence of a provirus with a large deletion encompassing the *pX* region may affect the interpretation of PVL measurements and viral gene expression analyses.

Regarding the effects of long-term passaging, integration site-specific PCR demonstrated integration sites A, C, and D in both MT-1 J and MT-1 M cells, indicating that these sites are conserved. By contrast, integration sites B and E were not detected in MT-1 M cells, suggesting that the composition of proviral integration sites changes during long-term culture, even in cell lines derived from the original clone, but maintained in different laboratories. These observations are consistent with previous findings in MT-2 cells and do not contradict earlier studies [9]. The differences in clonal frequencies observed between MT-1 J and MT-1 M may result from stochastic drift during long-term culture. Alternatively, certain proviral structures may confer a selective advantage, leading to shifts in clonal composition. However, because growth rates and selective pressures during serial passaging were not directly assessed, these interpretations remain speculative. It is also possible that MT-1 M may reflect an earlier clonal composition of the MT-1 lineage; however, this interpretation remains speculative.

Consistently, comprehensive analysis using RAISING identified integration sites A, C, and D as major proviral clones in MT-1 cells, in agreement with the results obtained by IL-PCR and I-PCR. By contrast, integration site E, which was detected by I-PCR, was not identified by RAISING. Because RAISING involves the synthesis of single-stranded DNA using HTLV-1-specific primers spanning the provirus-host junction, the absence of primer annealing sites in the provirus at site E may account for this discrepancy. In addition, methodological biases inherent to different detection approaches, including those related to fragment size, library preparation, and detection sensitivity, may also influence the observed integration site profiles [16]. RAISING identified several integration sites that were not detected by IL-PCR or I-PCR, indicating that the proviral clonal composition of MT-1 cells is non-uniform and the detected integration site profiles may vary depending on the analytical method and culture conditions.

This study has several limitations. Because IL-PCR and I-PCR are PCR-based methods, mutations or deletions in primer binding sites or restriction enzyme recognition sites may reduce detection sensitivity. In addition, differences in sensitivity and applicable fragment sizes among analytical methods indicate that no single approach can comprehensively identify all proviral integration sites. Future studies incorporating whole-genome sequencing approaches may enable more comprehensive analyses. Furthermore, as our analysis used only the MT-1 cell line, the findings cannot be directly generalized to other ATL cell lines without parallel analyses. Proviral copy numbers vary among commonly used ATL cell lines: HuT102 harbors 4–6 copies per cell, TL-Om1 harbors approximately 1.78 copies per cell, and ST-1 harbors approximately one copy per cell [11, 24, 25]. Previous studies have also demonstrated that Tax and HBZ expression levels differ depending on the HTLV-1-infected cell line examined [26]. Structural diversity of the HTLV-1 provirus and clonal variation arising during serial passaging may likewise differ among cell lines. Consequently, comparative analyses using multiple ATL cell lines will be necessary to determine whether the observations in the present study represent phenomena broadly shared among ATL cells. Systematic characterization of proviral copy number, integration sites, internal proviral structures, and viral gene expression profiles in widely used ATL cell lines will be particularly important for interpreting experimental findings in HTLV-1 research.

In conclusion, this study provides a comprehensive structural and clonal characterization of MT-1 sublines, a widely used ATL cell line, and demonstrates their polyclonal nature and heterogeneous proviral composition, including both full-length and defective HTLV-1 proviruses. Although proviral heterogeneity has been previously described in other HTLV-1-infected cell lines, such as MT-2 [9]. The present study extends these observations by demonstrating substantial variability in proviral composition among independently maintained MT-1 sublines. These findings have practical implications for experimental design and the interpretation of results obtained with MT-1 cells, particularly in studies of viral gene expression, proviral load, host gene regulation near integration sites, and the functional properties of infected cells.

## Data Availability

The original contributions presented in the study are included in the article. Further inquiries can be directed to the corresponding author.
